# A Skull-Removed Chronic Cranial Window for Ultrasound and Photoacoustic Imaging of the Rodent Brain

**DOI:** 10.3389/fnins.2021.673740

**Published:** 2021-05-31

**Authors:** Xuanhao Wang, Yan Luo, Yuwen Chen, Chaoyi Chen, Lu Yin, Tengfei Yu, Wen He, Cheng Ma

**Affiliations:** ^1^Department of Electronic Engineering, Tsinghua University, Beijing, China; ^2^Department of Ultrasound, Beijing Tiantan Hospital, Capital Medical University, Beijing, China; ^3^Beijing National Research Center for Information Science and Technology, Beijing, China; ^4^Beijing Innovation Center for Future Chip, Beijing, China

**Keywords:** cranial window, mouse model, ultrasound imaging, photoacoustic imaging, functional imaging

## Abstract

Ultrasound and photoacoustic imaging are emerging as powerful tools to study brain structures and functions. The skull introduces significant distortion and attenuation of the ultrasound signals deteriorating image quality. For biological studies employing rodents, craniotomy is often times performed to enhance image qualities. However, craniotomy is unsuitable for longitudinal studies, where a long-term cranial window is needed to prevent repeated surgeries. Here, we propose a mouse model to eliminate sound blockage by the top portion of the skull, while minimum physiological perturbation to the imaged object is incurred. With the new mouse model, no craniotomy is needed before each imaging experiment. The effectiveness of our method was confirmed by three imaging systems: photoacoustic computed tomography, ultrasound imaging, and photoacoustic mesoscopy. Functional photoacoustic imaging of the mouse brain hemodynamics was also conducted. We expect new applications to be enabled by the new mouse model for photoacoustic and ultrasound imaging.

## Introduction

Ultrasound imaging (USI) and photoacoustic imaging (PAI) are two rapidly developing imaging technologies for neuroscience (Xu and Wang, 2006; Xia et al., 2014; Moran and Thomson, 2020). USI visualizes internal brain structures via sound reflection (Rabut et al., 2019), while Doppler signals convey important functional information about blood flow (Demene et al., 2016). PAI, which exploits the optical absorption contrast and ultrasound detection, is potentially capable of imaging brain function by detecting signals from calcium or voltage sensors (Nasiriavanaki et al., 2014; Gottschalk et al., 2020). Moreover, by scanning the wavelength of the excitation light, multispectral PAI is sensitive to color thus providing a viable means for measuring the saturation of blood oxygen (Mallidi et al., 2009; Li et al., 2018). In the past, photoacoustic microscopy (PAM) and photoacoustic computed tomography (PACT) have been developed to provide a wide range of image resolution, depth, and field of view (FOV), offering a rather scalable imaging modality for various applications.

However, both USI and PAI suffer from a significant reduction in signal quality when ultrasound signals pass through the skull. The high acoustic impedance mismatch between skull and soft tissue (or ultrasound coupling gel) results in a great portion of the incident acoustic wave being reflected; the remaining sound wave that enters the skull is attenuated, scattered, and reflected again intricately, leaving a significantly attenuated and distorted transmitted wave (Gerstenmayer et al., 2018). The problem become even worse when the incident longitudinal wave is converted into shear mode, and when the cranial cavity induces reverberation of the signal. Acoustically, USI suffers a worse situation than PAI due to the round-trip signal path, yet in PAI, a reduction of signal strength is caused by the blockage of the excitation light by the skull (Yang et al., 2016).

Several computational approaches have been developed to model and correct the acoustic wave distortion in transcranial propagation (Odabaee et al., 2018; Mohammadi et al., 2019; Manwar et al., 2020). Kneipp et al., estimated the skull insertion loss using a photoacoustic point source and found good quantitative agreement between their model and measurements (Oraevsky et al., 2016). Yao et al. (2015) investigated the evolution of the acoustic waveform when penetrating through the skull (Liang et al., 2019). Acoustic ray-tracing was used to calculate the phase distortion along the acoustic travel path, and compensation algorithms were developed based on vector space similarity model and ray-tracing simulations (Mohammadi et al., 2020). Methods were developed to time-reverse the recorded temporal acoustic waveform, providing corrections for both phase distortions. Angular-spectrum-based method was also designed to model the effects of shear waves in transcranial imaging (Kozacki and Falaggis, 2015). Despite the extensive research, existing computational methods for skull aberration correction are not accurate enough, and are still not suitable for in-vivo imaging applications.

In recent years, experimental efforts for reducing the influence of the skull were proven effective. Heo et al. (2016) designed a soft, transparent, freely accessible cranial window for chronic imaging and electrophysiology. Park et al. (2015) implanted a cranial window on mouse cortex to study microvascular changes. Zhu et al. (2013) developed a number of optical clearing agents for mouse skull (Wan et al., 2018; Zhao et al., 2018), forming a switchable optical clearing window suitable for cerebrovascular imaging (Zhu et al., 2013; Zhang et al., 2018a). The acoustic clearing effect of the agents were also verified through in-vivo PAM imaging experiments (Liu et al., 2013). The methods above are applicable to optical imaging instead of USI or PAI. Li et al. (2019) developed a disposable chronic cranial window capable of ultrasound sensing, and demonstrated its effectiveness using longitudinal investigations by PAM.

Here we propose a mouse model with a long-term hidden cranial window, which is suitable for longitudinal studies with USI and PAI. The cranial window is simply created by removing the skull and suturing the incision on the scalp. After the wound completely heals, the created window can effectively eliminate a large portion of the signal attenuation and distortion from the skull. The animal with the acoustic window can resume its normal activities, and shows no sign of any health or mental conditions. Several imaging modalities were adopted to verify the effectiveness of the model. PACT can form images with deep-tissue optical absorption contrast, and is fast enough for brain function imaging (Cai et al., 2019). Photoacoustic mesoscopy provides a smaller field of view, yet it generates three-dimensional images with higher resolutions. Ultrasound and Doppler ultrasound imaging were also used to verify the mouse model, providing structural and functional information, respectively. We observed dramatically increased signal strength and improved image quality through a series of experiments conducted using two dimensional (2D) grayscale US, Doppler US, PACT, and three dimensional (3D) photoacoustic mesoscopy. To further verify the effectiveness of the model, functional cerebral hemodynamic changes in cortical blood vessels in response to either side of forepaw stimulation was also observed in cerebral coronal plane using the PACT system.

## Materials and Methods

### Animal Model

A specific pathogen free (SPF) mouse (CD-1 Nude, male, 8 weeks old, offered by Beijing Vital River Laboratory Animal Technology Co., Ltd.) was used for the modeling operation. A hand-held cranial drill with assorted drill head (size: 1.4 mm, round head, offered by RWD Life Technology Co. LTD) was used as surgical tools. All experimental procedures described here were in accordance with the National Institutes of Health Guidelines on the Care and Use of Laboratory Animal of Beijing Vital River Laboratory Animal Technology Co., Ltd. The operation was divided into four steps.

#### Step 1

**FIGURE 1:**
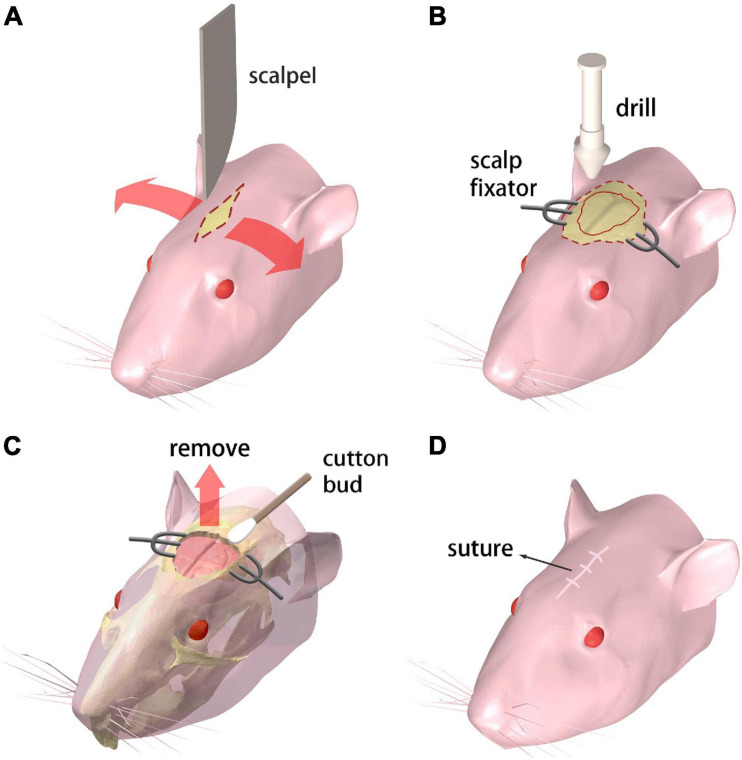
Procedure for creating the chronic cranial window. **(A–D)** Step 1 through 4, respectively. Refer to text for details.

The mouse receives the abdominal injection of Ketamine and toluene thiazide solution for anesthesia. Then the mouse is held in supine position on the operation desk which is heated to maintain the mouse body temperature. A 10 mm incision is cut on the scalp with a scalpel along the midline of the brain (Figure 1A).

#### Step 2

A pair of scalp fixators are used to separate the scalp along the incision. The meninges are ripped along the sagittal suture. Then a sterile swab is used to gently scrape the meninges off the skull for maximum bone exposure. A cranial drill is used to drill from the underside of the coronal suture The skull is gradually drilled thinner and furrowed along the coronal seam, and care is taken to prevent the skull from being drilled through. Then the drill moves down along the herringbone with the same drilling operation. Afterward, the drill moves along the sides of the skull, generating a closed-circle groove with a diameter of 10 mm (Figure 1B).

#### Step 3

A point hole is gently drilled at any point of the groove made in the previous step. Then an ophthalmic tweezer is used to remove the skull and expose the brain. Great care needs to be taken to avoid any damage to the meninx and the cerebral cortex. A sterile cotton swab is applied around the wound repeatedly to stop bleeding from the capillary network beneath the skull (Figure 1C).

#### Step 4

The scalp fixators are removed and the scalp is sutured with disinfection (Figure 1D). Subcutaneous injection of 20% (volume fraction) Tolfedine solution is operated with a reference dose of 0.1 ml/20 g bodyweight after the craniotomy is completed. This procedure is carried out to protect the brain tissue from scratching due to the wound on the skull and scalp.

The mouse is then bred in a dedicated cage alone to avoid unexpected injuries. The wound heals completely in 3 days. The stitches are removed after sustained recovery for 7 days and the mouse is ready for imaging experiments. Each mouse with the cranial window was imaged together with another normal mouse of the same age and weight as a control. Note that the processed mice are identical to the normal ones in terms of experimental handling. The cranial window is permanently created and protected by the scalp without disturbing the mouse’s activities and brain functions.

### PACT *in vivo* Imaging

**FIGURE 2:**
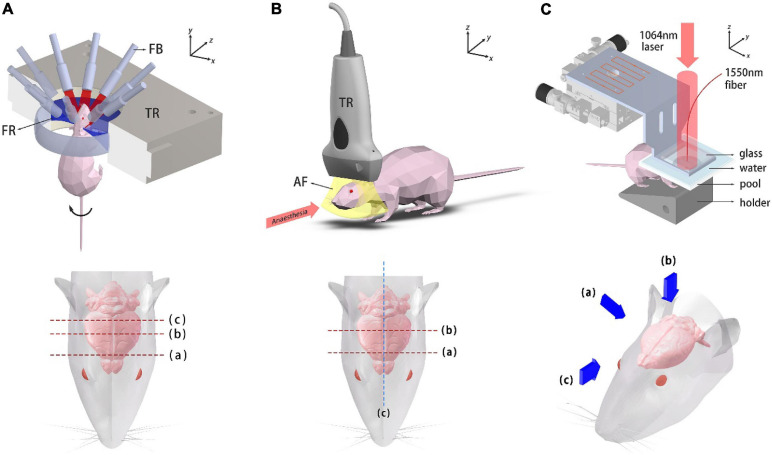
Schematic diagrams of the imaging systems. **(A)** Ring-array PACT system and top view of the mouse head. Dashed lines label the imaged coronal plane positions. TR, transducer array; FB, fiber bundle; FR, focusing region. **(B)** System setup of the USI system and top view of the mouse head. Dashed lines label the imaged coronal plane positions. TR, transducer probe; AF, acoustic focal plane. **(C)** Photoacoustic mesoscopy system and three viewing perspectives, which are presented with blue arrows.

A simplified illustration of the PACT system and imaging locations/perspectives are given in Figure 2A. A custom-made 256-element full-ring ultrasound detector array (Imasonic Inc.; 5.5 MHz central frequency; >60% −6 dB bandwidth) is used for photoacoustic signal detection. Each element is geometrically focused in the elevational direction to provide acoustic sectioning. The outputs from the detectors are fed into two 128-channel data acquisition units (Ultrasonix Medical, SonixDAQ; 40 MHz sampling rate; 12-bit ADC; 36–51 dB programmable gain) for A/D conversion. 10 Hz laser pulsing is synchronized with data acquisition. A 3D-printed holder was made to fix the mouse. In order to double the spatial sampling density, mechanical rotation is applied to the mouse holder, causing the holder to rotate back and forth at ±2π/512 (rad) between adjacent data acquisition. Two successive sinograms acquired at the interleaved positions are then merged. As for the PACT brain imaging experiment, an optical parametric oscillator (OPO) (SOLAR LP604,680–1064 nm tuning range, 10 Hz repetition, 10 ns temporal width) was employed. An excitation light wavelength of 850 nm was used for PACT brain imaging and the maximum energy density on the tissue surface (11 mJ/cm^2^ at 850 nm) was below the ANSI limits (100 mJ/cm^2^ at 1064 nm, 10 Hz laser pulse repetition rate). The light beams were expanded and subsequently coupled into a customized 1-to-10 fiber bundle (Silica fiber, transmission band 500–2400 nm). The distal ends of the fiber bundle were arranged such that on the imaged object an annular illumination pattern was formed on the focal plane of the transducer array. The system was controlled by a high-precision delay generator (Stanford Research Systems, DG645). A water circulation unit with temperature control modules was integrated into the system to maintain a relatively constant water temperature at around 31°C, keeping the animal in a normal state.

### Ultrasound *in vivo* Imaging

A schematic diagram of the USI system and the imaged locations are shown in Figure 2B. 1.5% isoflurane-air mixture is continuously supplied for anesthesia. The mouse was laid flat on the board which was heated to maintain its body temperature. A linear ultrasound probe was adopted for imaging (i24LX8, 8–24 MHz, Canon Corp.) which was connected to a clinical ultrasound machine (Aplio i800, Canon Corp.). The acquisition frame rate was 35fps for gray-scale structural imaging and 16 fps for color Doppler imaging.

### Photoacoustic Mesoscopic *in vivo* Imaging

The photoacoustic mesoscopy setup is schematically shown in Figure 2C. Briefly, a 1064 nm laser (LOTIS, LS-2145-LT150, pulse duration: 25 ns, repetition rate: 10 Hz) was used for PA excitation. The output beam was expanded and illuminated the mouse brain. The beam spot profile was shaped by two mirror reflections. A high-finesse Fabry-Pérot interferometric sensor was mounted on a raster-scanning stage with micrometer translation resolution in both *x* and *y* directions. The mouse was fixed onto a customized 3D-printed holder. A water tank whose bottom was covered with a polyethylene film was placed on top of the mouse head for acoustic coupling (with ultrasound coupling gel in between). A square grid of 128 × 128 sampling points were scanned and image reconstruction was subsequently performed in 3D.

### Functional PACT Imaging

We also used our PACT system to visualize the cerebral hemodynamic changes of the mouse subject to forepaw stimulation. A customized animal holder was used to fix the mouse stably. A 0.5 mm diameter transparent polytetrafluoroethylene (PTFE) tube was suspended vertically, 6 mm from the mouse head and was filled with diluted ink. The mouse was mildly anesthetized with 1% isoflurane-air mixture gas during the imaging experiment. A pair of bent-headed tweezers was employed to nip the forepaws as stimulations. An excitation light wavelength of 850 nm was used and for real-time imaging considerations, the full-ring ultrasound detector array stayed still during the imaging process. In each imaging plane, resting-state images were first acquired and by stimulating the left and right forepaw, another two sets of PACT images were acquired subsequently. A 90 s time gap was kept between stimulations to ensure full recovery of the mouse from the previous stimulation. In the whole imaging process, the temperature of water in the cavity was maintained approximately at the mouse body temperature.

### Data Processing and Image Reconstruction

For the PACT system, 131 frames of data were averaged with respiratory gating. Then a notch filter was applied to the channel data for denoising. Half-time delay-and-sum (DAS) method was adopted for image reconstruction (Anastasio et al., 2005). Half-time reconstruction was used to reduce artifacts generated by acoustic heterogeneity. In photoacoustic mesoscopy imaging, a 3D DAS algorithm was used to reconstruct the images. Hilbert transform was applied along the z axis to render the image unipolar, making it more realistic for 3D visualization. Dual-speed-of-sound was employed during image reconstruction to account for acoustic heterogeneity.

In the functional PACT imaging experiment, the lasers ran at 10 Hz. The pulse-to-pulse energy fluctuation of the laser was normalized using the PA signal of a PTFE tube placed in the field of view. For each frame, the data from the half-ring facing the mandible was unused. Full-time DAS reconstruction method was adopted and frames with respiratory motion were removed. By subtracting the PACT image without forepaw stimulation from the PACT images with forepaw stimulation, maps of cerebral hemodynamic changes were obtained.

## Results

### Animal Safety and Health Status Verification

**FIGURE 3:**
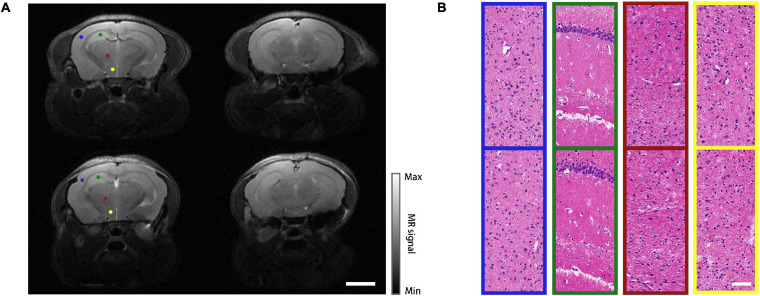
MRI and histopathology results. **(A)** MRI images of a normal mouse (top row) and a modeled mouse (bottom row), left column shows midbrain, right column shows hindbrain. Scale bar = 3 mm. **(B)** H&E stained images of different regions in midbrain coronal plane. Top row: normal mouse; bottom row: mouse model. The sampling regions of these images are represented as color-coded dots in **(A)**. Scale bar = 80 μm.

The proposed modeling approach has been implemented on 30 mice, out of which 22 survived after the operation (approximately 70% success rate). The leading cause of death was the large area of subcranial capillary hemorrhage during the operation. During an observation period of 6 weeks, the survived mice quickly regained normal physical activities and body weights. To better evaluate the health status of the mice, magnetic resonance imaging (MRI, T2 mode, PharmaScan 70/16 US, Germany, BRUKER) was performed on a modeled mouse and a normal one as a control. The representative midbrain and hindbrain images were shown in Figure 3A. Despite a slight cerebral cortical protrusion observed within the cranial window, the structure of the entire brain remained normal. Then the mice were euthanized for histopathology, in which thin sections of the brains were stained with hematoxylin and eosin (H&E) and investigated under a microscope. Comparison between the modeled mouse and the control were shown in Figure 3B. Four representative cerebral regions, i.e., frontoparietal cortex (FrPT), hippocampus (Hipp), thalamencephalon (Th) and hypothalamus (HyTh) were presented in different color boxes, which correspond to the color-coded dots marked on the MRI images in Figure 3A. No cellular structural lesion was found in the sections of the mouse model, indicating that the mouse was in good health status.

### PACT Imaging Results

**FIGURE 4:**
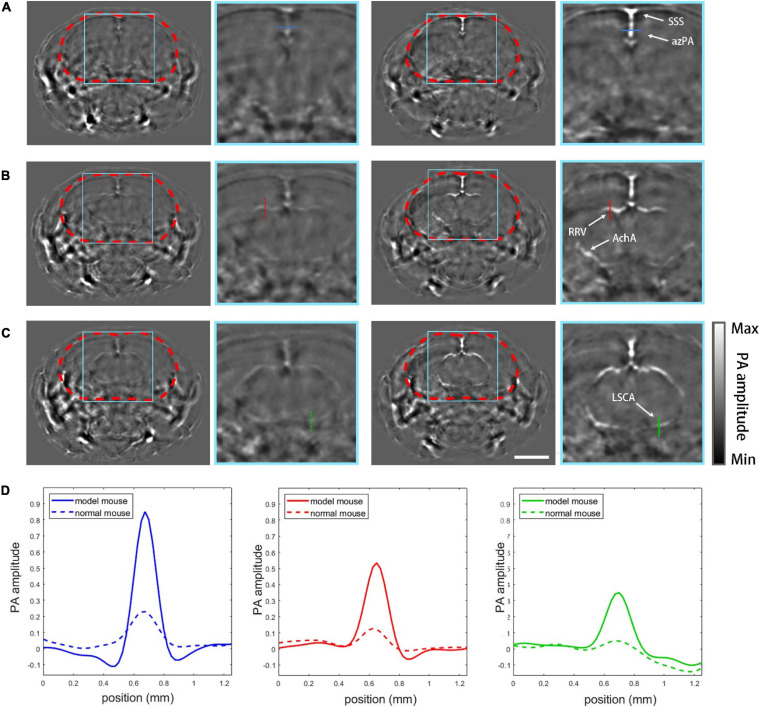
PACT imaging results. **(A)** PACT images of plane (a, Figure 2A) with zoomed-in views of normal mouse (left column) and the mouse model (right column). SSS, superior sagittal sinus; azPA, azygos pericallosal artery. **(B)** Images of plane (b, Figure 2A): RRV, rostral rhinal vein; AchA, anterior choroidal artery. **(C)** Images of plane (c, Figure 2A): LSCA, lateral superior cerebellar artery. **(D)** Profiles of the vessels marked in the PACT images by different colors. Scale bar = 3mm.

The effectiveness of the model was validated by the PACT imaging experiment. 20 coronal plane slices was acquired from frontal lobe to mid brain with a step of 250 μm, and 3 typical layers were shown with the zoomed view of the cranial cavity (Figure 4). For Figures 4A–C, the left group shows PACT imaging results of the normal mouse while the right group shows the skull-removed mouse model at the same imaging plane for comparison. Apparent on the PACT images, the profiles of the superior saggital sinus (SSS) and the azygos pericallosal artery (azPA) can be seen in the mouse with the cranial window, but the normal mouse shows very weak and blurry vessel features due to the absorption and distortion induced by the skull (Figure 4A). In Figure 4B the rostral rhinal vein (RRV) and the anterior choroidal artery (AchA) are clearly shown in the mouse with the window, but appeared fuzzy in the normal mouse. AchA almost completely disappeared in the image of the normal mouse. The lateral superior cerebellar artery (LSCA) showed similar distinctions between the tested mouse and its control counterpart (Figure 4C). The cross-sectional profiles of four selected vessels (azPA, RRV, AchA, and LSCA) are co-plotted with their corresponding control group in different line types (Figure 4D). Obviously, the vessel signal of the skull-removed mouse was about four times stronger than that of the normal mouse, which is consistent with an earlier theoretical prediction (Liang et al., 2019).

### Ultrasound Imaging and PA-US Dual Modality Imaging Results

**FIGURE 5:**
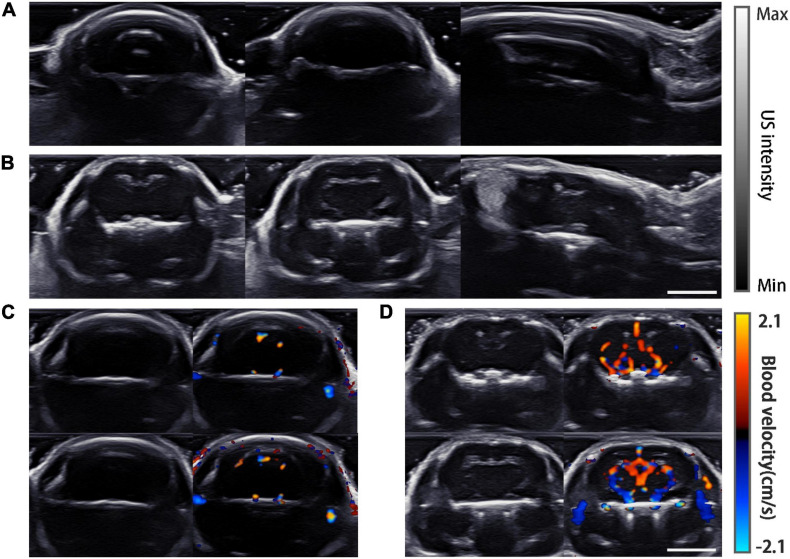
USI imaging results. **(A)** Gray-scale images of a normal mouse, from left to right are plane (a–c) which are shown in Figure 2B. **(B)** Gray-scale images of the mouse model imaged at the same planes as in **(A)**. **(C)** Doppler images of the normal mouse. Gray-scale images are displayed on the left. Two different layers are shown in the top and bottom rows. **(D)** Doppler images of the mouse model at the same planes of **(C)**. Scale bar = 5 mm.

Ultrasound images in the coronal and sagittal planes were acquired (Figure 5). The gray-scale ultrasound structural images show strong reflection artifacts for the normal mouse, while inside the cranial cavity all images were almost feature-less (Figure 5A). In comparison, the images of the mouse model show no reflection artifacts, and some internal features can be recognized in all the imaging planes (Figure 5B). For further verification, color Doppler flow imaging was carried out in two different coronal planes. Obviously in the normal mouse no Doppler signal was detected (Figure 5C) while the Doppler signals were strong in the processed mouse (Figure 5D).

**FIGURE 6:**
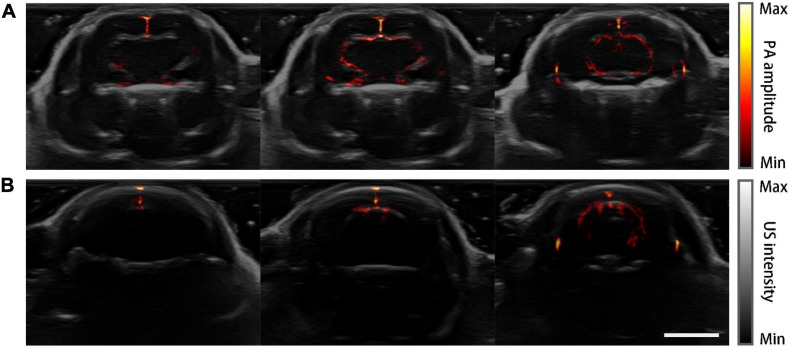
PA-US dual-modality imaging results. PA images are in pseudo-color and the US images are in grayscale as background. **(A)** PA-US images of the mouse model acquired at three coronal planes. **(B)** PA-US images of the normal mouse in the same planes of **(A)**. Scale bar = 5 mm.

Figure 6 shows the PACT images overlaid on top of the corresponding grayscale ultrasound images at the three representative planes. Drastic improvement of feature richness can be observed by comparing the images of the skull-removed and the normal mouse.

### Photoacoustic Mesoscopic Imaging Results

**FIGURE 7:**
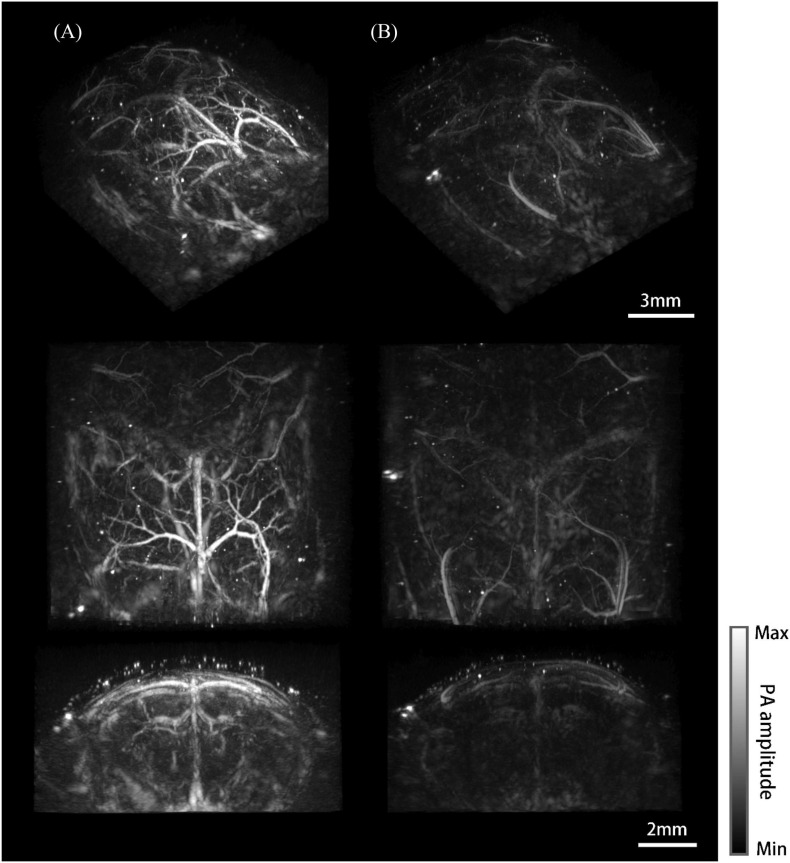
Photoacoustic mesoscopy system and imaging results. **(A)** Whole-brain imaging results of the mouse model. From top to bottom are the maximum intensity projections (MIP) of the viewing perspective (a–c) shown in Figure 2C, respectively. **(B)** Imaging results of the normal mouse.

Three-dimensional whole-brain imaging results of the mouse model and the normal mouse were reconstructed and compared. Different viewing perspectives are provided for each case (Figure 7). Figure 7A shows the imaging results of the mouse model. Big vessels of the brain, such as the superior sagittal sinus, azygos pericallosal artery, great cerebral vein of Galen and their side branches were clearly visualized (Dorr et al., 2007; Xiong et al., 2017). The Willis’ circle consisted of basilar vessels can be seen at a depth of 5 mm under the skin. It is worth mentioning that deep vessels are depicted with low resolution and CNR due to the reduced detection numerical aperture, dimmer excitation light, and larger breathing motion. In comparison, only the superior sagittal sinus and transverse sinus were barely visible and the coronal cavity seemed almost empty for the normal mouse (Figure 7B).

### PACT Imaging Results of Functional Cerebral Hemodynamics

**FIGURE 8:**
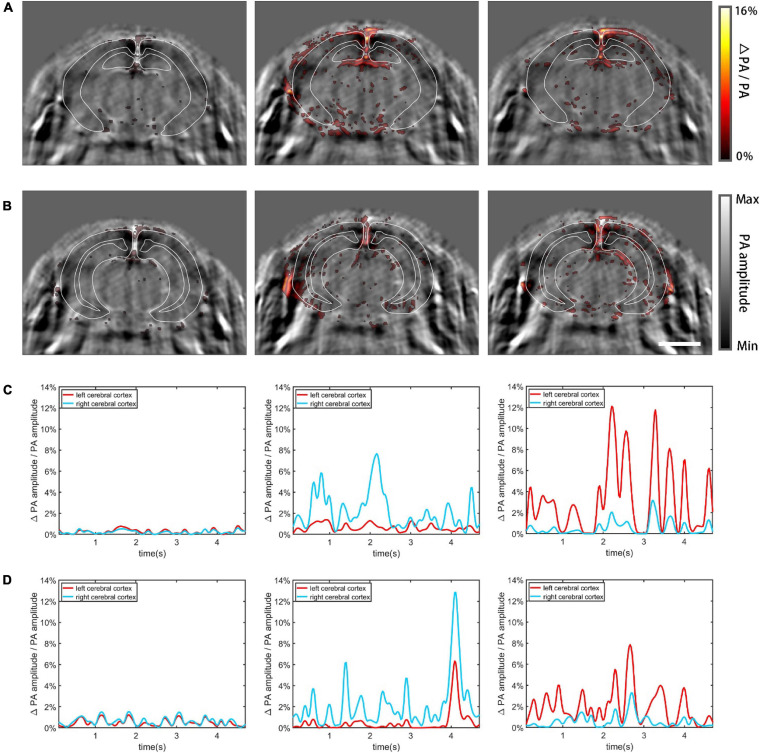
Functional cerebral hemodynamic PACT imaging results. The functional hemodynamic changes are in pseudo-color and the structural images are in grayscale as background. **(A)** Functional PACT images of the midbrain corresponding to: no external stimulation (left), left forepaw stimulation (middle), and right forepaw stimulation (right). **(B)** Functional PACT images of the hindbrain in the three cases as in panel **(A)**. **(C)** Relative change of the total signal in the midbrain cerebral cortex corresponding to the three different cases as in panel **(A)**. **(D)** The same plots as in panel **(C)** for the hindbrain. Scale bar = 3 mm.

Increased neural activity causes enhanced local cerebral blood flow and regional metabolic activity (Dowling et al., 1996; Gerrits et al., 1998; Jones et al., 2002). Using the mouse model, we imaged functional cerebral hemodynamic changes in different coronal planes in response to forepaw stimulation (Zhang et al., 2018b). According to the PA imaging result, the brain regions that exhibited enhanced signals following the stimulation matched the cerebrovasculature well (Figures 8A,B), proving that the detected functional signals resulted from hemodynamic changes. Figure 8A shows in pseudo color the proportional signal change of the midbrain, superimposed on gray scale structural PA images. Three different cases are shown from left to right: no external stimulation, left forepaw stimulation, and right forepaw stimulation, respectively. The observed dynamic patterns are similar to previously published results (Avanaki et al., 2013; Yao et al., 2015) where the contralateral side of the brain lit up, indicative of increased activity due to the stimulation. In Figure 8A, the cerebral cortex was segmented using white solid lines based on the Allen Mouse Brain Common Coordinate Framework (Wang et al., 2020). Figure 8C plots the relative change of the total signal in the segmented cortex regions, corresponding, respectively, to the PA images above. The graphs clearly show that the two hemispheres exhibit significantly different responses to unilateral forepaw stimulation. The results of hindbrain PACT imaging are shown in Figures 8B,D. Movies of the above results are provided in Supplementary Material.

## Discussion

The effectiveness of the mouse model was proved by ultrasound and photoacoustic imaging, both in structural and functional imaging. For PACT imaging, the main vascular branches were clearly visualized in the mouse model, while the features were much weaker and blurrier in the normal mouse. The functional PACT imaging results revealed the cerebral hemodynamic changes in response to forepaw stimulation, showing the potential for in-depth studies of specific regions of the brain (Liao et al., 2010). The usefulness of the model was further validated by ultrasound imaging where ultrasound signals have to penetrate the skull twice. Although we only tested the most basic ultrasound imaging functions, the conclusion is applicable to other USI techniques implementing various sequences. The benefit from the subcutaneous cranial window is expected to be more pronounced in high-resolution ultrasound or photoacoustic imaging with more advanced scanning and image processing methods applied. This is well-proven in the PA mesoscopy experiment where, due to the wider bandwidth and angular reception of the PA signal, imaging without the skull made a dramatic difference. Besides enhancing image quality, the new model is very easy to make—only a common craniotomy is needed without any complex operations or extra materials. The model is superior for longitudinal studies, and imaging the new model is as convenient as imaging a normal mouse.

We believe that the new model is favorable compared to the traditional intravital thinned skull approach (Yang et al., 2010). The skull thinning procedure produced local heating, and surgeries are needed before every imaging experiment (Holtmaat et al., 2009). Although it was reported that craniotomy can potentially cause glymphatic dysfunction, gliosis, and changes in neurologic functions due to exposure infections and intracranial pressure imbalance (Plog et al., 2019), there are abundant evidences that craniotomy is safe for experimental animals (Askoxylakis et al., 2017; Cha et al., 2020). Based on a 3-month observation, the mice with the hidden cranial windows lived a normal life after the operations and had active physiological responses to external stimuli. Further, the modeled mouse regained body weights soon after the surgery, which clears any potential biosafety issues. Since no chemical agents are involved to construct the model, the risks of infection, toxicity, and other side effects are low. Thus, we believe the mouse model reported in this article is suitable for a wide range of imaging applications involving ultrasound excitation and detection.

We have succeeded in implementing the technique to various categories of experimental mice, including nude mice (CD-1 Nude, Balb/c Nude) and common laboratory mice (CD-1, Balb/c), and all experiments confirmed our conclusion above. Currently, the cranial window mainly covers the top of the mouse skull. It is more desirable if the window can be extended to partially open the sides of the skull. However, this is expected to perturb the normal activity and pose health risks. Future studies are needed to decide the size limit of the window with ensured biosafety.

## Data Availability Statement

The raw data supporting the conclusions of this article will be made available by the authors, without undue reservation.

## Ethics Statement

The animal study was reviewed and approved by National Institutes of Health Guidelines on the Care and Use of Laboratory Animal of Beijing Vital River Laboratory Animal Technology Co., Ltd.

## Author Contributions

XW proposed the mouse modeling methods and conducted all *in vivo* experiments. YL assisted in all the *in vivo* experiments. YC performed data processing and participated in the photoacoustic mesoscopic imaging experiments. CC participated in the functional PACT imaging experiment and involved in its data processing. LY and TY participated in the ultrasound imaging experiment. WH and CM oversaw the project. All authors wrote and edited the manuscript.

## Conflict of Interest

The authors declare that the research was conducted in the absence of any commercial or financial relationships that could be construed as a potential conflict of interest.
