# Supplementary material for: A Skull-Removed Chronic Cranial Window for Ultrasound and Photoacoustic Imaging of the Rodent Brain
Source: Front Neurosci. 2021 May 31;15:673740. doi: 10.3389/fnins.2021.673740 (PMC8200560; doi:10.3389/fnins.2021.673740)
Supplement: Supplementary file 1 [file Table_1.DOCX]

Supplementary Material

**Supplementary Movie 1.** Functional cerebral hemodynamic PACT imaging results of the midbrain corresponding to no external stimulation.

**Supplementary Movie 2.** Functional cerebral hemodynamic PACT imaging results of the midbrain corresponding to left forepaw stimulation.

**Supplementary Movie 3.** Functional cerebral hemodynamic PACT imaging results of the midbrain corresponding to right forepaw stimulation.

**Supplementary Movie 4.** Functional cerebral hemodynamic PACT imaging results of the hindbrain corresponding to no external stimulation.

**Supplementary Movie 5.** Functional cerebral hemodynamic PACT imaging results of the hindbrain corresponding to left forepaw stimulation.

**Supplementary Movie 6.** Functional cerebral hemodynamic PACT imaging results of the hindbrain corresponding to right forepaw stimulation.
